# Reply to: Comparison of bronchial hygiene techniques in mechanically ventilated patients: a randomized clinical trial

**DOI:** 10.5935/0103-507X.20190081

**Published:** 2019

**Authors:** Wagner da Silva Naue, Bruno Barcelos Herve, Fernando Nataniel Vieira, Gracieli Nadalon Deponti, Luciane de Fraga Martins, Alexandre Simões Dias, Silvia Regina Rios Vieira

**Affiliations:** 1 Hospital de Clínicas de Porto Alegre, Universidade Federal do Rio Grande do Sul - Porto Alegre (RS), Brasil.

**To the Editor**

The authors would like to express their gratitude for the comments and for the opportunity to clarify the details of the study published in the *Revista Brasileira de Terapia Intensiva* .^(1)^ With respect to the perception that aspiration alone was more effective than pressure-controlled hyperinflation, it is important to note that hyperinflation was performed with an inspiratory time of 1.67 seconds and an inspiratory/expiratory ratio of 1/2 (as described in the methodology) to decrease the peak inspiratory flow relative to the peak expiratory flow and to avoid auto-PEEP. In addition, while studies in the laboratory using lung simulators and animal models suggest that volume-controlled ventilation is more effective for hyperinflation and control of the inspiratory rise time,^(2,3)^ these models differ greatly from the sample of our study. Previous studies used ventilatory modes in which patients were ventilated with pressure-supported ventilation,^(4,05)^ thus justifying the choice of this ventilatory mode for hyperinflation.

The randomization of the study may have caused some confusion; however, the study was not a crossover clinical trial. The crossover of aspiration with the hyperinflation technique (hyperinflation with mechanical ventilator, vibrocompression and hyperinflation with mechanical ventilator associated with vibrocompression) was performed only in the first 24 hours, according to the methodology described in the study, to evaluate the outcome of aspirated pulmonary secretion. After this period, the selected hyperinflation technique was performed, and the patients underwent mechanical ventilation twice a day until extubation or death.

We also emphasize that additional studies comparing different ventilatory modes of pulmonary hyperinflation in patients under mechanical ventilation are necessary to clarify this point.
